# The Significant Effects of Threshold Selection for Advancing Nitrogen Use Efficiency in Whole Genome of Bread Wheat

**DOI:** 10.1002/pld3.70036

**Published:** 2025-01-21

**Authors:** Mohammad Bahman Sadeqi, Agim Ballvora, Said Dadshani, Md. Nurealam Siddiqui, Mohammad Kamruzzaman, Ahossi Patrice Koua, Jens Léon

**Affiliations:** ^1^ INRES‐Plant Breeding Rheinische Friedrich Wilhelms University Bonn Germany; ^2^ INRES‐Plant Nutrition Rheinische Friedrich Wilhelms University Bonn Germany; ^3^ Department of Biochemistry and Molecular Biology Bangabandhu Sheikh Mujibur Rahman Agricultural University Gazipur Bangladesh; ^4^ Field Lab Campus Klein‐Altendorf Rheinische Friedrich Wilhelms University Bonn Germany

**Keywords:** bread wheat, FDR, GWAS, linear and nonlinear algorithms, threshold, whole genome

## Abstract

Currently in wheat breeding, genome wide association studies (GWAS) have successfully revealed the genetic basis of complex traits such as nitrogen use efficiency (NUE) and its biological processes. In the GWAS model, thresholding is common strategy to indicate deviation of expected range of *p*‐*value*(s), and it can be used to find the distribution of true positive associations under or over of test statistics. Therefore, the threshold plays a critical role to identify reliable and significant associations in wide genome, while the proportion of false positive results is relatively low. The problem of multiple comparisons arises when a statistical analysis involves multiple simultaneous statistical tests, each of them has the potential to be a discovery. There are several ways to address this problem, including the family‐wise error rate and false discovery rate (FDR), raw and adjusted *p*‐*value*(s), consideration of threshold coherence and consonance, and the properties of proportional hypothesis tests in the threshold definition. We encountered some limitations in the definition of FDR threshold, particularly in the upper bounds of linear and nonlinear approaches. We emphasize that empirical null distributions based on permutation test can be useful when the assumption of linear or parametric FDR approaches do not hold. Nevertheless, we believe that it is necessary to utilize modern statistical optimization techniques to evaluate the stability and performance of our results and to select significant FDR threshold. By incorporating the neural network algorithm, it is possible to improve the reliability of FDR threshold and increase the probability of identifying true genetic associations while minimizing the risk of false positives in GWAS results.

**Abbreviations**
FDR: false discovery rate
FWER: family‐wise error rate
GWAS: genome wide association studies
LD: linkage disequilibrium
LFDR: local FDR
MAF: minor allele frequency
MCMC: Markov chain Monte Carlo
NUE: nitrogen use efficiency
QTL: quantitative trait locus
SNP: single nucleotide polymorphisms

## 1 Introduction

Bread wheat ( *Triticum aestivum* L.) is one of the most important cereals and covers the food requirements of a large part of the world's population. In general, nitrogen use efficiency (NUE) of bread wheat is estimated to be only 30%–40% of the total amount of nitrogen fertilizer applied that is actually harvested, although genotype and environment (G × E) interaction and cropping practices have a significant impact on NUE in bread wheat and significant proportion of phenotypic variance is genetically based (Cormier et al. 2016; Salim and Raza 2019). NUE is considered as complex trait for breeding and is controlled by many genes with small effects or small *p*‐*value*(s). In wheat breeding, genome‐wide association studies (GWAS) have successfully revealed the genetic basis of complex traits and biological processes (Uffelmann et al. 2021; Rathan et al. 2022). Genetic parameters such as population structure, genomic relationship matrix, marker density, sample size and minor allele frequency (MAF) have major impact on the power and accuracy of GWAS model (Wang et al. 2016). However, they play crucial role in estimating the threshold for the significant false discovery rate (FDR). In GWAS, one side of the model is assigned to a complex trait such as NUE. However, to reduce multicollinearity and heteroscedasticity in the NUE observations, model evaluation and error measurement is required. Furthermore, thresholding is a common strategy in the GWAS model to indicate the deviation from the expected range of *p*‐*value*(s) with respect to single nucleotide polymorphisms (SNP) under or over the test statistic. Thus, the threshold plays a crucial role in identifying significant features in wide genome as possible, while at the same time, a relatively low proportion of false positives occurs (Storey 2002). In traditional GWAS models, linkage disequilibrium (LD) was tested locus by locus with traditional *p*‐*value* cutoff 0.01 or 0.05 as threshold to minimize false positives. LD analysis at each locus with probability of *p*‐*value* = 0.05 for false positives is stringent and conservative criterion because very few loci in the results show significant linkage (Zabaneh and Mackay 2003) and it seems unlikely in a large association panel. The advent of high‐throughput sequencing technologies and the availability of large‐scale genomic data have enabled researchers to investigate GWAS models with various complex quantitative traits. As a result, multi‐locus mixed GWAS models by emphasis on large numbers of hypotheses with small effects have been proposed (Segura et al. 2012; Chen, Liu, and Xie 2016). The problem of multiple comparisons arises when a statistical analysis involves multiple simultaneous statistical tests, each of which has the potential to be a discovery. There are different ways to control this problem, for example, the family‐wise error rate (FWER) and FDR, raw and adjusted *p*‐*value*(s), consideration of threshold coherence and consonance, and properties of proportional free and restricted hypothesis tests in threshold definition.

### 1.1 FDR Thresholding Based on Linear Approaches

*Bonferroni* correction based on frequentist statistical inference is common method to handle FWER, which is proper for experimental studies such as quantitative trait locus (QTL) analysis. The *Bonferroni* correction has an intensive significance level (α) of the null hypothesis to control the likelihood probability of false positive in multiple hypothesis testing (Haynes 2013). The *Bonferroni* correction is a simple and conservative technique with low power. This adjustment depends on the sample size and does not affect range of effect sizes. Moreover, in this adjustment, maximum likelihood (ML) of both type I and type II errors increase, which is making the most GWAS results significant but they are not truly existent (Perneger 1998; VanderWeele and Mathur 2018). *Holm* adjustment is another frequentist method to deal with FWER that attempts to estimate the ML for the threshold line in multiple comparison hypotheses. It simultaneously covers ML estimation and tow way analysis of variance (Giacalone et al. 2018; Lee and Lee 2020), which is appropriate for genome wide case and control studies, not for association panel studies. *Hochberg* adjustment is an alternative approach to determine the smallest FWER significant level and is less sensitive than *Bonferroni* correction to deal with ML of FWER (Tan and Xu 2014; Chen, Feng, and Yi 2017). The idea of thresholding with adjusted *p‐value*(s) instead of raw *p‐value*(s) was first proposed in the *Benjamini‐Hochberg* approach (Reiner, Yekutieli, and Benjamini 2003). Generalization of adjusted *p‐value*(s) from raw *p‐value*(s) in this approach is complex to compute (Krzywinski and Altman 2014). Threshold based on adjusted *p‐value*(s) has the potential to minimize error in FDR estimation, resulting in the detection of marginal null hypotheses from the peaks. Depending on the number of marginal null hypotheses that are true, threshold line in the *Benjamini‐Hochberg* approach can be less conservative than the *Bonferroni* correction (Benjamini and Bogomolov 2013; Brinster et al. 2018). *Benjamini‐Yekutieli* approach may be a first attempt to incorporate the concept posterior distribution of FDR into threshold line definition. In practice there are many extreme raw *p‐value*(s) even smaller than 2.7e−09 in the GWAS results, which is a limitation to estimate FDR with high accuracy. To deal with many simultaneous confidence intervals for FDR, it is less powerful than *Benjamini‐Hochberg* approach (Furmańczyk 2013; Brinster et al. 2018). Like the *Bonferroni* correction, *Sidak* adjustment is also appropriate to deal with FWER threshold when small number of hypotheses are correlated (Blakesley et al. 2009). The origin of this correlation lies in genetic parameters such as MAF and the level of LD in the genomic file. In *Sidak* adjustment, null hypotheses are classified using stepwise FWER controlling procedure, while threshold estimation is based on fixed bounds of raw *p‐value*(s) (Midway et al. 2020).

### 1.2 FDR Thresholding Based on Nonlinear Parameters

Currently, the threshold is largely considered a linear parameter in the differentiated GWAS models, for example, single locus or multi locus association (Cook, Mahajan, and Morris 2016; Wen et al. 2017; Lozano et al. 2023). Thresholding based on linear estimator has only mean squared error as scale for high dimensional genomic datasets. In general, scaled version of adjusted *p‐value*(s) in GWAS results does not simply follow the distribution of raw *p‐value*(s), and it is risky to soft thresholding. The first attempts to avoid this risk were to consider the threshold as nonlinear concept (Wilson 2019; McCaw et al. 2022; Asif et al. 2020), and *q‐value*(s) was introduced as an alternative approach to the adjusted *p‐value*(s) method for thresholding. The *q‐value*(s) is still widely used in genome wide studies and corresponds to the expected proportion of false positive results rather than the ML estimate of false positive rate (Storey and Tibshirani 2003). While the estimator plays an important role in the FDR thresholding, the main drawback of this nonlinear estimator is that the *q‐value*(s) is a random variable and FDR can be underestimated, which can lead to unexpected false positives (Lai 2017; Menyhart, Weltz, and Győrffy 2022). Also, based on raw *p‐value*(s), the *q‐value*(s) increases with the function $\pi_{0}\left(\lambda \right)=1$. This function controls the proportion of raw *p‐value*(s) used for the null distribution (*z‐value*(s)). However, λ is a nonlinear parameter with a value 0 to 1. Even by bootstrap or permutation techniques, the λ closer to 1 implies increasing variance in $\pi_{0}$, leading to autocorrelation and heteroscedasticity at the FDR thresholding. The second attempt to avoid the risk of soft thresholding in large‐scale simultaneous hypothesis testing was the *local* FDR (LFDR) threshold (Efron 2013); *LFDR* features tail regions of the null distribution and provides both scaling and computational power for large scale genome wide studies. For the given adjusted *p‐value*(s), *LFDR* measures the posterior probability of the local false positives using *empirical Bayesian* (EBayes) algorithm (Efron 2013; Korthauer et al. 2019). All three adjustments *q‐value*(s), $\pi_{0}\left(\lambda \right)$, and *LFDR* are computed based on the proportion of false positives from the adjusted *p‐value*(s). It is assumed that these comparisons are independent. However, the mean square error of the comparisons is affected by the outliers in the adjusted *p‐value*(s), especially if there are strong negative correlations between the comparisons simultaneously.

### 1.3 FDR Threshold Optimization

In FDR thresholding, poor generalization performance is the main problem in genome wide datasets with large‐scale null hypotheses, simultaneously, and the generalization algorithms require more attention (Sørensen et al. 2022). While some studies confirmed that threshold is a nonlinear parameter concept (Emmert‐Streib and Dehmer 2019; Cao, Sun, and Yao 2023), we believe that sparsity and scale of false positives in the large simultaneous hypothesis tests are two important features in the FDR approach. The sparsity assumption in FDR threshold implies that there are only a small number of true positive associations in GWAS results with non‐zero SNP effects (Hastie, Tibshirani, and Wainwright 2020). The scaling assumption is a helpful technique, when error minimization is objective, as it separates the hierarchical false positives from true positives. Both sparsity and scaling assumptions lead to soft thresholding with smoother null distribution and hard thresholding where the peaks and likelihood median of the posterior distribution are preserved, using *Gibbs* sampling based on the Markov chain Monte Carlo (MCMC) algorithm. FDR optimization is particularly useful for hard and soft thresholding, when sparsity and scale of large simultaneous hypothesis are considered in the definition and selection of threshold. Depending on the wide genome dataset, the location of threshold and the posterior mean or median threshold for large simultaneous hypothesis tests are still a problems in threshold selection. Therefore, in GWAS, determining the significance threshold that separates true and reliable associations from random noises required more revision of approaches. However, genetic parameters and hyper‐parameters have significant impacts on the definition and optimization of FDR threshold (Chen, Robinson, and Storey 2019). In this study, we evaluate different thresholding approaches and their performance to optimize false positive rates in the context of a phenotypic and genotypic NUE dataset of 221 bread wheat genotypes. We emphasize that focusing on the threshold definition and its statistical inference could be able to determine significant threshold with reliable results. Therefore, the objectives of this study are to compare distinguished FDR thresholding approaches: (i) identify an appropriate regularization parameter for the given FDR thresholding approach, (ii) optimize genetics parameters and hyper‐parameters in the given FDR thresholding approach, and (iii) demonstrate the performance of the best thresholding approach through empirical Bayes coherence behavior and compliance estimation.

## 2 Results

### 2.1 Phenotypic Quality Control

Combined ANOVA by considering the N level, year, and replication as fixed effect showed that there was a significant difference between low, middle, and high N applications. Also, there was a significant difference between the genotypes as random factor within 3 years. However, the effect of N × G was significant for NUE, and its related traits include GY, GNY, SY, and SNY (Table 1). Therefore, the non‐significant N × G × Y = 75 interaction indicates that the NUE observations can be utilized as a phenotypic vector in the given GWAS model.

**TABLE 1:** Combined analysis of variance of NUE and its agronomic‐related traits at low and high N levels, during 2018, 2019, and 2020.

SOV	*df*	GY	GNY	SY	SNY	NUE
Y	2	2,478,000***	360***	1751***	0.0070***	1067***
N	1	1,380,000,000***	3523***	211,535***	1.4513***	130,883***
G	441	225,800,000***	39***	69,902***	0.6343**	112,752***
N × G	882	411,700^ns^	10***	705^ns^	0.0048***	430***
N × Y	2	170,500,000***	13***	144,976***	0.1227***	45,862***
G × Y	220	785,200***	1.40^ns^	1422***	0.0025^ns^	104^ns^
N × G × Y	440	562,700^ns^	1.41^ns^	1107^ns^	0.0029*	75^ns^

*Note:* Mean squares for NUE and its agronomic related traits are shown.

Abbreviations: df: degree of freedom; GNY: grain nitrogen yield (%); GY: grain yield (kg/ha); NUE: nitrogen use efficiency (%); ns: not significant; SNY: straw nitrogen yield (%); SOV: source of variation; SY: straw yield (kg/ha).

* Significant at 0.05 level.

** Significant at 0.01 level.

*** Significant at 0.001 level.

**FIGURE 1:**
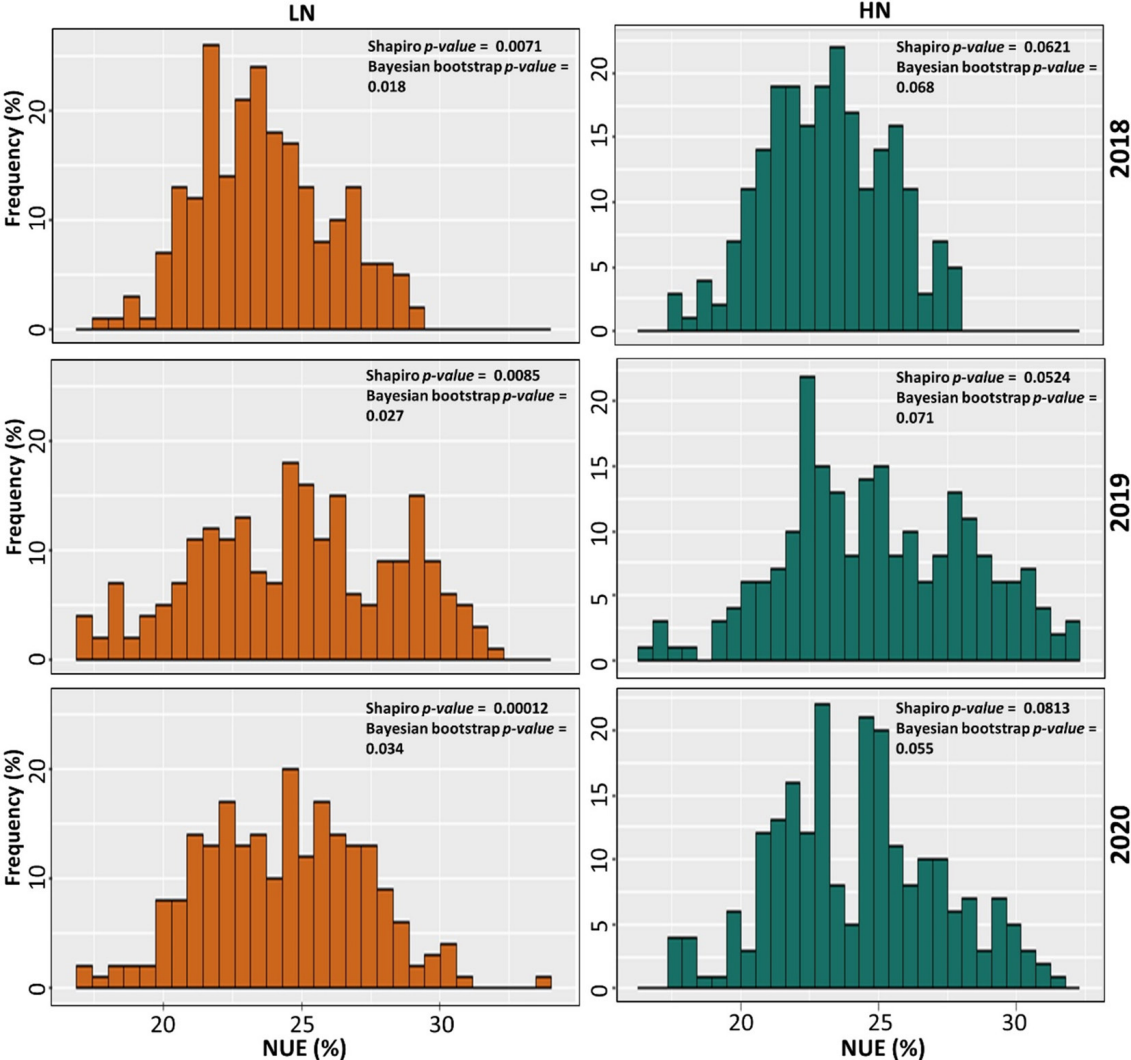
NUE vector quality control via Shapiro *p‐value* and Bayesian bootstrap *p‐value*; both *p‐values* refer acceptable deal with outliers in the vector at low and high N levels, 2018, 2019, and 2020. The mean Bayesian bootstrap *p‐value* = 0.0263 for low N and the mean Bayesian bootstrap *p‐value* = 0.0641 for high N are close to 95% CI of mean Shapiro *p‐value*(s), which verifies distribution of NUE vector is normal.

To address outliers in the NUE vector associated with 221 bread wheat genotypes evaluated over three years, the data was averaged across these years, resulting in a mean value of 24.181 (%) with a standard error of 11.16 (%). In the histogram plot (Figure 1), the mean of Shapiro *p‐value* = 0.0307, and the mean of Shapiro *p‐value* = 0.0652 for low and high N, respectively, across 3 years, which indicates the NUE vector is following normal distribution. We assumed that this test is not enough to decide on normality of NUE vector, so to focus more on the quality of observations, 2000 times the repeated random vectors with replacement from original NUE vector was generated. The mean Bayesian bootstrap *p‐value* = 0.0263 and the mean Bayesian bootstrap *p‐value* = 0.0641 for low and high N are close to two‐tailed 95% confidence interval (CI) of mean Shapiro *p‐value*(s), which confirms the distribution of NUE vector is normal.

### 2.2 Genomic Quality Control

**FIGURE 2:**
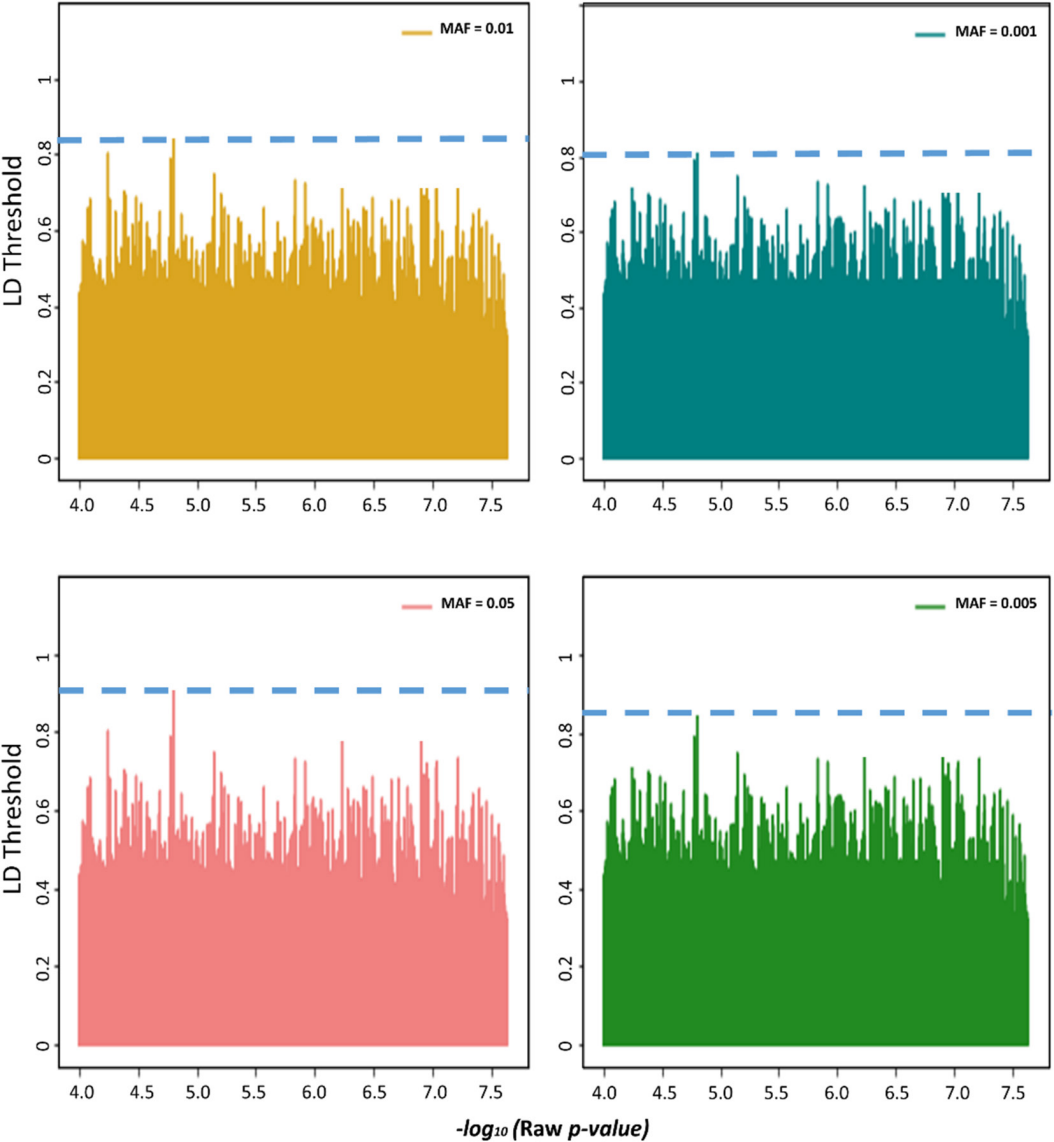
Visualization‐fixed MAF values less than or equal to 0.001, 0.005, 0.01, and 0.05 to the platform of 150K affymetrix chip with 22,489 SNP polymorphic among 221 bread wheat genotypes. LD threshold was specified as dash line for each MAF values. With MAF ≤ 0.05 and –log_10_ (*p‐value*) = 4.75, the highest value of LD threshold was received.

Applying various fixed MAF values less than or equal to 0.001, 0.005, 0.01, and 0.05 to the platform of 150 K affymetrix chip with 22,489 SNP polymorphic among our 221 bread wheat genotypes, we found as expected at MAF ≤ 0.05, and at *–log*_*10*_ *(p‐value)* = 4.75, we would have the highest value of LD threshold ($r^{2}$ = 0.95) (Figure 2). While, with the other MAF values, the LD thresholds are still high ($r^{2}$ ≥ 0.80), it seems that genomic datasets with very low MAF have low heterozygosity and are then less informative. Therefore, this genome‐wide LD threshold could be utilized in the given GWAS model and the linear FDR approaches such as Bonferroni correction for 150 K SNP variants with MAF ≥ 0.05 and the FDR line *–log*_*10*_ *(p‐value)* ≅ 5.

### 2.3 Generation of Adjusted *p‐value*(s)

**FIGURE 3:**
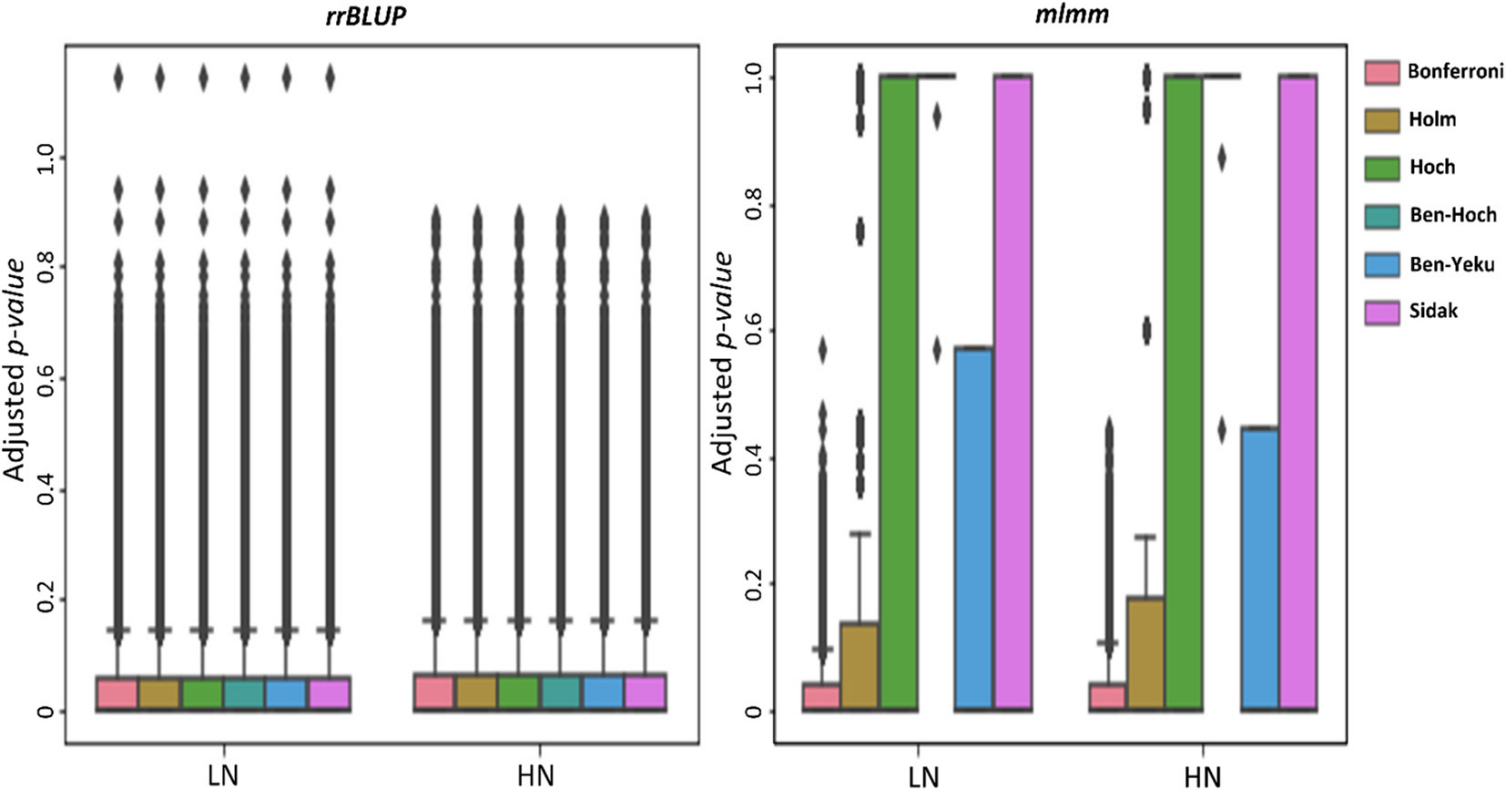
The impact of raw *p‐value*(s) for NUE vector, received from left: single locus (*rrBLUP*) and right: multi locus associations (*mlmm*) GWAS models, under low and high N for *Bonferroni* correction, *Holm*, *Hoch*, *Benjamini‐Hochberg*, *Benjamini‐Yekuteili*, and *Sidak* adjustments, was investigated, respectively. Both *Hoch* and *Sidak* approaches cover very well all range of raw *p‐value*(s) for NUE vector received from *mlmm* GWAS model, which implies to acceptable approaches to adjust the raw *p‐value*(s) at low and high N levels.

The impact of raw *p‐value*(s) for NUE vector received from single locus (*rrBLUP*) and multilocus associations (*mlmm*) GWAS models under low and high N was revealed (Figure 3). Our findings on adjusted *p‐value*(s) show that, based on *rrBLUP* model, the likelihood of NUE vector for all linear FDR thresholding approaches is equal to 0.06 at low and high N levels. Due to type I error and high number of false positives in the raw *p‐value*(s) produced by the *rrBLUP* GWAS model, the high bias in the adjusted *p‐value*(s) vector is observed. Therefore, this implies that the raw *p‐value*(s) received from this GWAS model could not be prone to use in the FDR thresholding approaches. In contrast, based on *mlmm* model, the likelihood of NUE vector is different depend to linear FDR thresholding approach. The *Bonferroni* correction and *Benjamini‐Hochberg* approaches might not cover all range of raw *p‐value*(s) received from *mlmm* GWAS model. In contrast, both *Hoch* and *Sidak* approaches have upper bounds $\frac{\alpha }{m-i+1}$ and $1-\frac{\alpha }{m}$, respectively, which minimize the bias in the FWER estimation. In addition, this linear approaches cover very well all range of raw *p‐value*(s) received from *mlmm* GWAS model, which implies to acceptable approaches to adjust the raw *p‐value*(s) at low and high N levels.

### 2.4 FDR Thresholding Based on Linear Approaches

**FIGURE 4:**
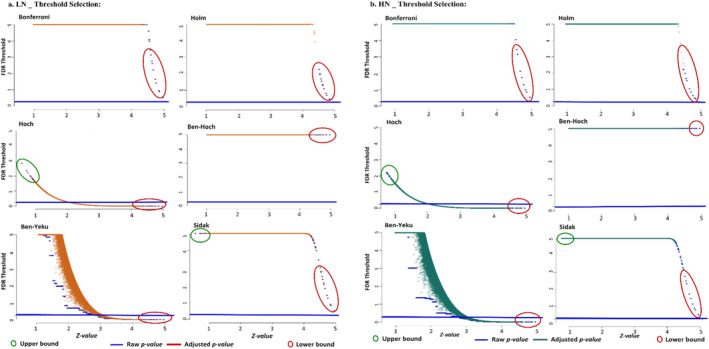
FDR thresholding based on the *Bonferroni* correction, *Holm*, *Hoch*, *Benjamini‐Hochberg*, *Benjamini‐Yekuteili*, and *Sidak* linear approaches among adjusted and raw *p‐value*(s) at low (a) and high (b) N levels. The performance of each FDR linear approach was highly dependent on the characteristics of the upper and lower bunds of their functions. Only the *Hoch* and *Sidak* linear adjustments in the upper and lower bounds could clearly provide false positives.

The performance of each FDR linear approach was highly dependent on the characteristics of the upper and lower bunds of their functions (Figure 4). The results based on the six distinguished FDR linear approaches demonstrated that FDR threshold line 0.05 could not be true value to make decision on false positives, which is common value in GWAS models. In addition, the results show that the scaled version of adjusted *p‐value*(s) does not follow easily distribution of raw *p‐value*(s) and it is risky to accept the results of this linear approaches. However, in all approaches at low and high N levels, ML of null hypotheses was defined as regularization parameter in the lower bund to detect false positives. In contrast, in the upper bound, only the *Hoch* and *Sidak* linear adjustments could clearly provide false positives, and we could assume that both related upper bounds $\frac{\alpha }{m-i+1}$ (in the *Hoch* adjustment) and $1-\frac{\alpha }{m}$ (in the *Sidak* adjustment) were defined proper to receive reliable and reproducible results in aim to FDR thresholding. The number of false positives in upper bound of both *Hoch* and *Sidak* adjustments are the same range. But when comparisons were extended to the lower bounds, the distribution of false positives was different.

### 2.5 FDR Thresholding Based on Nonlinear Approaches

**FIGURE 5:**
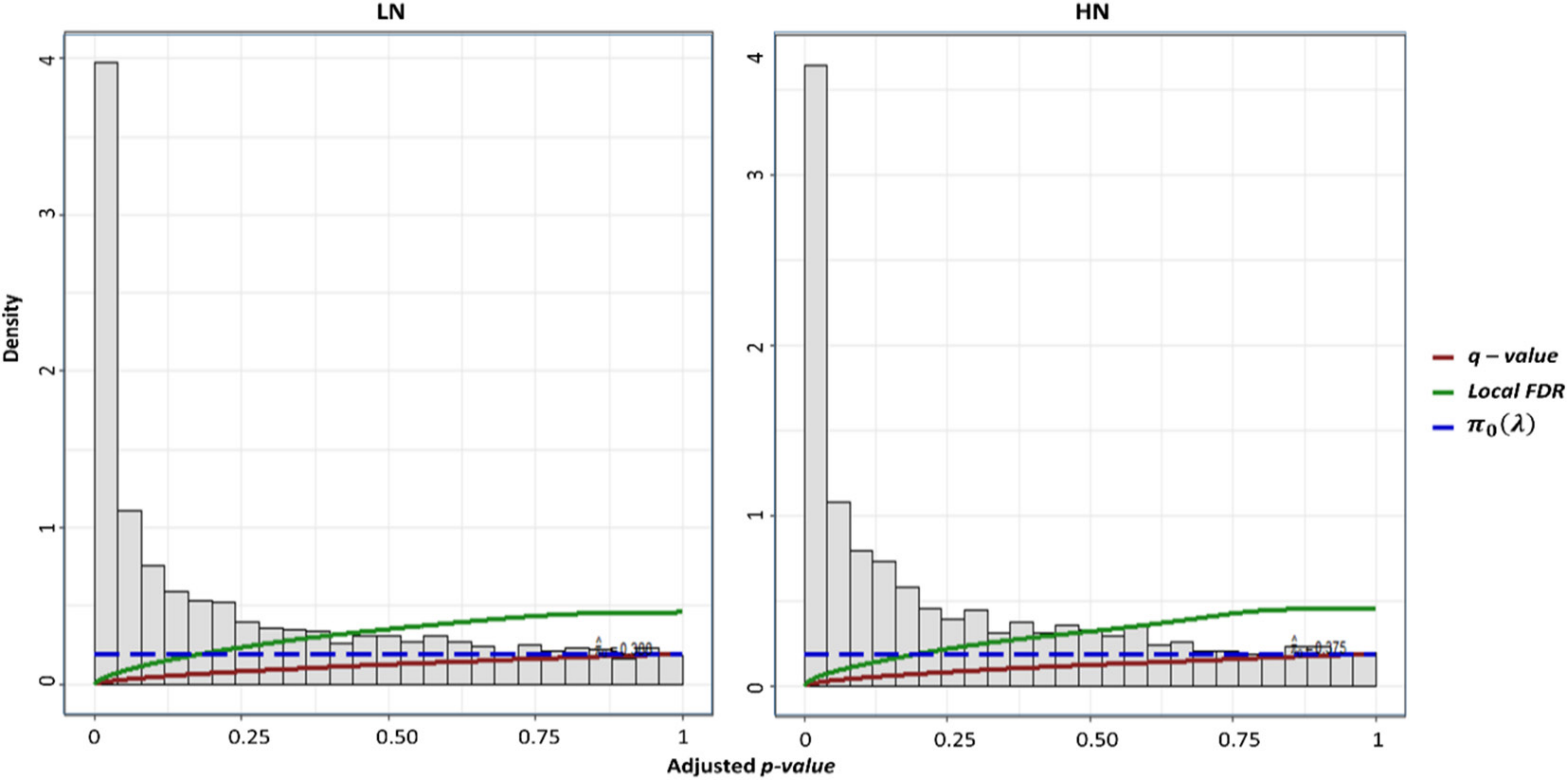
The performance of *q‐value*(s) and *LFDR* as distinguished nonlinear in the context of thresholding. Density of false positives in both methods using the adjusted *p‐value*(s) is same at low and high N levels, which is significantly reduced the risk of soft thresholding. Due to coincidence in the distribution tails, there is no significant difference in performance between *q‐value*(s) and $\pi_{0}\left(\lambda \right)$. *LFDR* in the upper tail area at low and high N levels shows coherent behave for the FDR threshold, which make it reasonable accurate upon large‐scale simultaneous problem.

We evaluated the performance of *q‐value*(s) and *LFDR* as distinguished nonlinear in the context of thresholding (Figure 5). An interesting finding is that the density of false positives in both methods, when using the scaled version of adjusted *p‐value*(s), is the same at both low and high N levels. This significantly reduces the risk of soft thresholding compared to FDR linear approaches. According to the definition, for both non‐linear approaches, the prior probability of association hypotheses was identified by estimating $\pi_{0}\left(\lambda \right)$ as regularization parameter. Due to coincidence in the distribution tails, there is no significant difference in performance between *q‐value*(s) and $\pi_{0}\left(\lambda \right)$ = 0.388 and 0.375 at low and high N levels, respectively. Due to genetic parameters and hyper‐parameters such as MAF, LD, and SNP high density, for the *LFDR* thresholding curve against $\pi_{0}\left(\lambda \right)$ = 0.388 and 0.375 at low and high N, there is no quite coincidence seems. In spite of no coincidence in the LFDR to $\pi_{0}\left(\lambda \right)$ distribution, $f\left(z_{i}\right)$ function in the definition could be estimated using EBayes algorithm. Therefore, *LFDR* in the upper tail area at both low and high nitrogen levels exhibits consistent behavior with the FDR threshold, making it reasonably accurate for large‐scale simultaneous problems.

### 2.6 FDR Threshold Optimization and Selection

**FIGURE 6:**
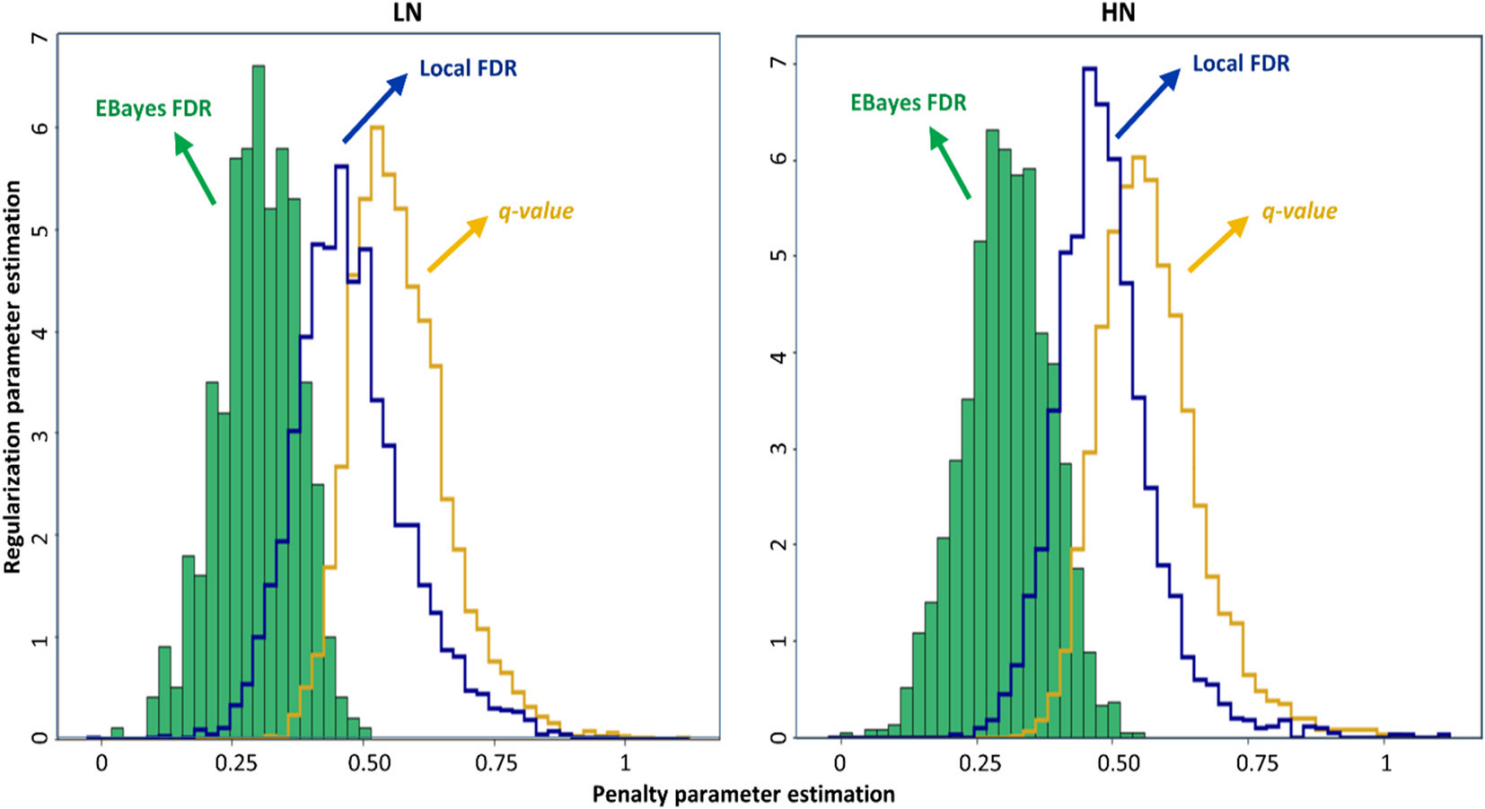
Posterior distribution estimation for *q‐value*(s) and local FDR thresholding approaches at low and high N using sparsity (with regularization parameter) and scaling (with penalty parameter) assumptions. In the solid area histogram: *EBayes* = 15,000 *Gibbs* samples with $\hat{\mu }$ = 5.8 and k = 0.25 at low N and $\hat{\mu }$ = 5.8 and k = 0.25 at high N were generated. For the local FDR with dark blue line histogram: $\hat{\mu }$ = 6.0 and k = 0.50 at low N and $\hat{\mu }$ = 6.9 and k = 0.50 at high N. For the *q‐value*(s) with orange line histogram: $\hat{\mu }$ = 5.9 and k = 0.60 at low N and $\hat{\mu }$ = 5.8 and k = 0.60 at high N.

To optimize the FDR threshold in the linear approaches, ML was estimated while it was faced with bias in the results. Even bias‐variance analysis could not minimized this negative effect on the heavily tail simultaneous distributions at all. However, this problem was observed in the *Benjamini‐Yekuteili* approach clearly and in the other linear approaches more or less. Consequently, the estimation of ML as lower bounds was with bias and upper bounds with high variance. Therefore, the bounds could not estimate the significant FDR threshold, and they were removed from optimization. Then, we evaluated the results of FDR nonlinear approaches that include *q‐value*(s) and *LFDR* using *EBayes* FDR as index with uninformative to mildly informative in the prior distribution of false positives (Figure 6). The *EBayes* false positives histogram was obtained with 15,000 Gibbs samples in two dimensions including regularization parameter ($\hat{\mu }$ = 5.8) for sparsity and penalty parameter (k = 0.25) for scaling optimization. The results show that posterior distribution in the *LFDR* might be a good demonstration in dealing with false positives especially in heavily tailed areas. The *LFDR* approach utilizes the cumulative distribution function of the given adjusted *p‐value*(s) in the tails as the prior distribution. This cumulative function optimizes the sparsity and the scale of false positives very well in contrast to the *q‐value*(s).

## 3 Discussion

In GWAS, genetic parameters such as population structure, genomic relationship matrix, marker density, sample size, and MAF have large effects on the performance and accuracy of the model (Wang et al. 2016). However, they play an important role in estimating the significant FDR threshold. In GWAS, one side of the model is allocated to the complex trait such as NUE. To reduce the multi‐collinearity and heteroscedasticity in the NUE observations, model evaluation and error measurement are necessary. In the combined ANOVA, consideration of the N level, year, and replication as fixed effects showed that there was a significant interaction between genotype and environment at low and high N levels. Therefore, NUE is considered as a complex trait to breed and is controlled by many genes with minor effects or small *p‐value*(s). In addition, the null hypothesis in the GWAS model often states that there is no association between a particular genetic variant and the trait of interest. The selection of significance threshold is a critical parameter used to identify reliable associations between genetic variants and complex trait. The significance threshold is set to control and minimize type I error rate. It has this potential to compute the probability of true null hypotheses that they are falsely rejected. It helps the researchers to distinguish true positive associations from random false positive associations with reasonable accuracy. In addition, the reliability and reproducibility of significant associations will be robust and applicable to different populations. The selection of significance threshold depends on various factors such as MAF, LD threshold, raw or adjusted *p‐value*(s), and the desired balance between sensitivity and specificity of FDR approach. Raw *p‐value*(s) represent the probability that a test statistic will have values as extreme as those obtained in the study. If the null hypothesis is true, it means there is no association between the genetic variant and the complex trait. The FDR thresholding based on raw *p‐value*(s) is affected by noise in the false positive results. Therefore, to improve the robustness and validity of the calculated FDR threshold, we used bootstrap technique to generate the adjusted *p‐value*(s) vector from the raw *p‐value*(s). To address this issue, we applied various FDR thresholds in linear forms including *Bonferroni* correction, *Holm*, *Hoch*, *Benjamini‐Hochberg*, *Benjamini‐Yekuteili*, and *Sidak* adjustment or in nonlinear forms including *q‐value*(s) and *local* FDR in platform of empirical Bayes estimation. While some reports have generally shown that FDR thresholding is a non‐linear concept (Emmert‐Streib and Dehmer 2019; Cao, Sun, and Yao 2023), we believe that sparsity and scale of false positives in the large simultaneous hypothesis tests are two important features. Sparsity refers to the proportion of true null hypotheses in a set of hypotheses to be tested. With high sparsity, when a large proportion of hypotheses are true nulls, the FDR threshold could be accurate. Our results confirm that linear approaches are associated with a less stringent FDR threshold, which may not be appropriate because the overall rate of false discovery is controlled even if a higher proportion of individual discoveries turn out to be false positives. However, in all FDR linear approaches at low and high N levels, the ML value of null hypotheses was defined as regularization parameter in the lower bund to detect false positives. However, ML estimation was associated with bias in the results. Even the bias‐variance analysis could not minimize this negative effect on the heavily tailed simultaneous distributions. In contrast, both *q‐value*(s) adjustment and *LFDR* are calculated based on the proportion of false positives through the function $\pi_{0}\left(\lambda \right)=1$. The scaling refers to the adjustment of the significance threshold based on the number of null hypotheses, the similarity in behavior between the distribution of adjusted *p‐value*(s) and raw *p‐value*(s), along with the complexity of the given GWAS model. However, adjusting the FDR threshold based on the scaling assumption is crucial when dealing with a large number of hypotheses to control the overall rate of false positives. In the context of FDR thresholding, the *EBayes* index could be utilized to predict the *q‐value*(s) and *LFDR* performances, which contain the prior information that a given test statistic corresponds to a null hypothesis given the genomic data. The *EBayes* usually includes shrinkage or smoothing of estimates, which could improve the stability *LFDR* estimates, especially when dealing with sparse data or low scaled where individual estimates may be unreliable due to small sample sizes. Heterogeneity often occurs in the distribution of true and null effects across different genomic features. However, FDR threshold results are accompanied by bias in the estimation. The *EBayes* algorithm could be designed to account for this heterogeneity, leading to more accurate *LFDR* estimates in the heavy tails of specific genomic regions. However, we evaluated the results of FDR nonlinear approaches including *q‐value*(s) and *LFDR* using the *EBayes* algorithm as powerful index with uninformative to slightly informative content in the prior distribution of false positives.

In summary, *q‐value*(s) and *LFDR* serve similar purposes in controlling false positives in high‐dimensional data but have different focuses and applications. The choice between them depends on the definition of approach, the objectives of analysis, and the characteristics of the data under investigation. The *q‐value*(s) provides a global control for the false positives, whereas the *LFDR* provides more reasonable information at the genotype test level. Therefore, in our study, in spite of no coincidence in the *LFDR* to $\pi_{0}\left(\lambda \right)$, the $f\left(z_{i}\right)$ function in the definition could be estimated using the *EBayes* algorithm. Moreover, *LFDR* shows coherent behavior for the FDR threshold in the upper tail region at low and high N levels, which makes it a reasonable approach, while other FDR thresholds cannot reveal very well the distribution of false positives in the tail regions. Furthermore, the interpretation of false positives in GWAS results requires some caution when using linear FDR adjustments in sparse and scale of genomic data. We encountered some limitations in defining FDR threshold, particularly in the upper bounds of linear and nonlinear approaches. We emphasize that empirical null distributions based on permutation method can be useful when the assumption of linear or parametric FDR approaches does not hold. Nevertheless, we believe that it is necessity to use modern statistical optimization techniques to evaluate the stability and performance of our results and to select significant FDR threshold. By incorporating neural network algorithm, it is possible to improve the reliability of the FDR threshold and increase the likelihood of identifying true genetic associations while minimizing the risk of false positives in GWAS results.

## 4 Method

### 4.1 Phenotypic Dataset

In this study, a set of 221 bread wheat genotypes received from breeding innovations in wheat for resilient cropping systems (BRIWECS) project were widely grown in agricultural research station Klein‐Altendorf, University of Bonn, 50°37′8.5″ N, 6°59′25.4″ E, for three cropping seasons 2017–2018, 2018–2019, and 2019–2020. In order to limit competition effects, all genotypes were sorted by maturity date and were planted in layout of split‐plot design, in two replication, six blocks, and 1326 plots. Each plot was 5 × 2.5 m^2^ consisting of 24 rows, 800 viable seeds of each genotype per m^2^. Seed rate and planting date were run according to common local practice. N treatment was taken main plots (factor A with fixed effect) and genotypes as sub‐plots (factor B with random effect). Every winter, to check soil homogeneity, before fertilizer application, soil sampling was performed in all blocks from three depths 0–30, 30–60, and 60–90 cm, and mineral‐N, ammonium‐N, and nitrate‐N were tested by LUFA laboratory, Nordrhein‐Westfalen, Germany. Two N levels, namely, 110 kg N ha^−1^ or low N (LN) and 220 kg N ha^−1^ or high N (HN), were applied as N treatment, and then related traits to NUE were recorded. NUE in wheat is defined as GY per unit of N supplied from soil or fertilizer (Moll, Kamprath, and Jackson 1982):

(4.1.1) $$\mathrm{NUE}=\frac{\mathrm{Gw}}{N_{s}}=\left(\frac{N_{t}}{N_{s}}\right)\left(\frac{\mathrm{Gw}}{N_{g}}\right)\left(\frac{N_{a}}{N_{t}}\right)\left(\frac{N_{g}}{N_{a}}\right)$$

where Gw is grain weight, $N_{s}$ is the nitrogen supply or rate of N fertilizer in the soil samples in depths 30, 60, and 90 cm, and $N_{t}$ is total N in the plant at maturity (grain + straw), with following formula in practice: $N_{t}=\left(\frac{\mathrm{GY}.\;\mathrm{GNC}}{100}\right)+\left(\frac{\mathrm{SY}.\;\mathrm{SNC}}{100}\right)$. So GY is grain yield, GNC is grain N concentration measured with near‐infrared spectroscopy (NIRS) machine, SY is straw yield, SNC is straw N concentration with NIRS machine, $N_{g}$ is equal to GNC or grain N concentration measured with NIRS machine, and $N_{a}$ is aboveground plant N accumulated after heading. To analyze N and genotypes as main effects and N × G, during three agronomic years, a combine analysis of variance (ANOVA) on grain yield (GY), grain nitrogen yield (GNY) with formula $\mathrm{GNY}=\left(\frac{\mathrm{GY}.\;\mathrm{GNC}}{100}\right)$, straw yield (SY), straw nitrogen yield (SNY) by $\mathrm{SNY}=\left(\frac{\mathrm{SY}.\;\mathrm{SNC}}{100}\right)$, and NUE were calculated using additive main effects and multiplicative interaction models (AMMI) with *R/agricolae*. To deal with outliers in the NUE vector, they were kept, because they were reflecting the actual field values across all years. The residuals distribution was checked using *Shapiro* normality test. To focus more on the quality of the vector, 2000 times the repeated random samples with replacement from original NUE vector was simulated using R/*bootstrap*, then Bayesian bootstrap *p‐value*(s) was calculated.

### 4.2 Genomic Dataset

In order to characterize NUE vector among 221 bread wheat genotypes, a platform of 150 K affymetrix SNP Chip at TraitGenetics GmbH (SGS GmbH Gatersleben, Germany) was used. After checking SNPs deviated from the Hardy–Weinberg equilibrium (HWE), only 22,489 polymorphic SNP markers were remained and used in GWAS model as genomic file (Sadeqi, Ballvora, and Léon 2023). The SNPs with MAF ≤ 0.05, ≤ 0.01, ≤ 0.005, and ≤ 0.001, respectively, were removed due to monomorphism in the marker (Fadista et al. 2016). To detect the regions that might be involved in LD, based on the pruned marker information, neighbored LD between adjacent SNPs ($D^{\prime }$) within 200 Mb with promised to physical position of SNPs on each chromosome and genetic correlation between two loci ($r^{2}$) with MAF value (Joiret et al. 2022) was calculated using R/*Synbreed*.

### 4.3 GWAS Models and Adjusted *p‐value*(s) Generation

GWAS single locus association model with R/*rrBLUP* was fitted in the adjusted form the mixed linear model as y=Xβ+Zu+e, where y is the trait vector, X is the fixed effects matrix, β is the vector of coefficients including principal components and population structure, Z is the matrix of random SNP effects coded as (−1, 0, and 1), $V\left(u\right)=K\sigma_{g}^{2}$, where K is the GRM as kinship matrix, and $\sigma_{g}^{2}$ is additive genetic variance with IBS basis. It was removed from the model due to convergence of N × Y to zero. GWAS multi‐locus association model was fitted with R/*mlmm.gwas* in form $\;y_{i=1}^{n}=\mu +\sum_{j=1}^{m}M_{.j}\beta_{j}+e$, where $\;y_{i=1}^{n}$ is NUE vector with n genotypes, m is total number of SNPs, $M_{\mathrm{ij}}$ is the matrix of random SNP effects coded as (0, 1 and 2), and $\beta_{j}$ is the vector of SNP effects and $H_{0}$ denoted in form of $\beta =\sigma_{g}^{2}=0$. Based on both GWAS models, after rejection of $H_{0}=\beta =0$ for each SNP, the vector *–log*_*10*_ *(raw p‐value(s))* was retrieved. Due to high type I error and high number of false positives in the raw *p‐value*(s) produced by the given GWAS model, it is uninformative and does not provide a reliable vector regarding FDR thresholding approaches. To enhance reliability and reproducibility of FDR approaches, it is necessity to adjust the raw *p‐value*(s). Therefore, proportion of null raw *p‐value*(s) with 2000 times bootstrap random SNPs (with replacement) from the original raw *p‐value*(s) vector was estimated using R/*fdrtool*. Using the bootstrapping technique, the hyper‐parameter $\left(\lambda =0.05\right)$ was tuned for linear threshold approaches. Similarly, using the smoothing technique, the hyper‐parameter $\left(\lambda =0.01\right)$ was tuned for non‐linear threshold approaches. Then based on given GWAS model (*rrBLUP* or *mlmm*) and through distinguished linear FDR thresholding approaches, the adjusted *p‐value*(s) vector at low and high N levels was generated (Boca and Leek 2018; Jafari and Ansari‐Pour 2019).

### 4.4 FDR Thresholding Based on Linear Approaches

For the FDR thresholding based on linear approaches, six common methods will be considered include following adjustments using R/*FDRestimation*:

1 *Bonferroni* correction is defined by the following function (Nakagawa 2004):

(4.4.1) $${\text{Bonf}}_{p-\text{value}}=\sum_{i=1}^{m}\mathrm{ML}\left(\mathrm{\pi i}\leq \frac{\alpha }{m}\right)$$

where ${\text{Bonf}}_{p-\text{value}}$ corresponded for Bonferroni correction *p‐value* generated from hypothesis tests, m is number of hypothesis tests, ML is maximum likelihood of multi comparisons when $H_{0}$ is rejected and a type I error is produced, πi is linear threshold coefficient factor for 𝑖th paired comparisons, and α is confidence interval of paired comparisons (usually 0.05). Bonferroni correction only accounts the number of $H_{0}$ tests, separately, while definition does not have a component to cover the relation between hypothesis tests and FWER, simultaneously.

2 *Holm* threshold adjustment utilizes the same function, which is in the Bonferroni correction; only instead of upper bound $\frac{\alpha }{m}$, the equality is in the definition, as follows (Giacalone et al. 2018):

(4.4.2) $${\text{Holm}}_{p-\text{value}}=\sum_{i=1}^{m}\mathrm{ML}\left(\mathrm{\pi i}=\frac{\alpha }{m}\right)$$

With this equality, the type II error increases lower than Bonferroni correction, but the other norms are getting same explanation.

3 *Hochberg* adjustment is defined with the function (Chen, Feng, and Yi 2017):

(4.4.3) $${\text{Hoch}}_{p-\text{value}}=\sum_{i=1}^{m}\mathrm{ML}\left(\mathrm{\pi i}\leq \frac{\alpha }{m-i+1}\right)$$

The Hochberg threshold adjustment conducts statistical inference of hypothesis by starting with the maximum *p‐value* from $H_{m}$ to $\;H_{i}$ and then to $H_{0}$. Moreover, due to upper bound $\frac{\alpha }{m-i+1}$, the *p‐value*(s) are taking weights. This weight vector minimizes bias in the FWER.

4 *Benjamini‐Hochberg* procedure controls the FDR using function (Benjamini and Hochberg 1995):

(4.4.4) $$\mathrm{Ben}-{\text{Hoch}}_{p-\text{value}}=\sum_{i=1}^{m}\mathrm{ML}\left(\mathrm{\pi i}\leq \frac{\alpha \left(m+1\right)}{2m}\right)$$

Due to upper bound $\frac{\alpha \left(m+1\right)}{2m}$ in the function, the ratio false discoveries to all discoveries at 0.01 significance leads to estimate the non‐false discovery rate (NFDR) in the procedure. However, the procedure has been constructed for adjusted *p‐value*(s).

5 *Benjamini‐Yekutieli* procedure controls the FDR using function (Benjamini and Yekutieli 2001):

(4.4.5) $$\mathrm{Ben}-{\text{Yeku}}_{p-\text{value}}=\sum_{i=1}^{m}\mathrm{ML}\left(\mathrm{\pi i}\leq \frac{\alpha \left(m+1\right)}{2m\left[\ln\left(m\right)+1\right]}\right)$$

Due to logit bound $\frac{\alpha \left(m+1\right)}{2m\left[\ln\left(m\right)+1\right]}$ in the function, and based on adjusted *p‐value*(s), the ratio of false positives to all discoveries at 0.01 significance might to find optimal value for threshold line.

6 *Sidak* adjustment is defined with the function (Chen, Feng, and Yi 2017):

(4.4.6) $${\text{Sidak}}_{p-\text{value}}=\sum_{i=1}^{m}\mathrm{ML}\left(\mathrm{\pi i}\leq 1-\frac{\alpha }{m}\right)$$

In the function, $1-\frac{\alpha }{m}$ is a complementary event to minimize the bias in the FWER.

### 4.5 FDR Thresholding Based on Nonlinear Approaches

For the FDR thresholding based on nonlinear approaches, two distinguished methods will be considered including:

1 *q‐value* threshold was utilized to minimize the error variance of threshold in the posterior distribution of adjusted *p‐value*(s) using the following function (Storey and Tibshirani 2003) using Bioconductor/*q‐value*:

(4.5.1) $$\hat{\text{qFDR}}=\text{minP}\left(\pi_{0}\left(\lambda \right)=0\;\right|z\in \left[c,\infty \right))$$

where qFDR corresponded for *q‐value* generated from function, P is posterior probability of type I errors when $\pi_{0}\left(\lambda \right)=0$ and $H_{0}$ is rejected, $f\left(c\right)$ is proper cumulative distribution of given adjusted *p‐value*(s) when $P\left(Z\geq c\right)$, and $f\left(z\right)$ is the distribution of null *z‐value*(s).

2 *Local FDR* was utilized to concentrate on tail‐area calculations over adjusted *p‐value*(s) with Bayesian inference, using the following function (Efron and Tibshirani 2002) using Bioconductor/*twilight*:

(4.5.2) $$\text{LFDR}=P\left(\left(\pi_{0}\left(\lambda \right)=0\;\right|Z=z_{i}\right)\;\text{if}\;\lambda =\frac{\pi_{0}f\left(z_{0}\right)}{f\left(z_{i}\right)}$$

where LFDR corresponded for FDR value generated from function, P is posterior probability of type I errors when $\pi_{0}\left(\lambda \right)=0$ and $H_{0}$ is rejected, $z_{i}$ is a t‐statistic comparing pairwise SNP associations with *N* (0, 1) distribution under null hypothesis, λ is proportion of true positives to all hypotheses, and $f\left(z_{i}\right)$ is calculated based on EBayes rule that is the posterior distribution estimate at median point.

### 4.6 FDR Threshold Optimization

Due to checking generalization performance in FDR thresholding, the optimization method including regularization and penalization is designed to determine significant threshold with high accuracy. In the given threshold approach, sparsity assumption has been measured with regularization parameter and scaling assumption with penalty parameter. In the FDR thresholding based on linear approaches, ML and upper bound of each function including (α,m) were taken as regularization parameter and penalty parameter, respectively. Also, for the FDR thresholding based on nonlinear approaches, $\pi_{0}\left(\lambda \right)$ and $f\left(z\right)$ of each function were taken as regularization parameter and penalty parameter, respectively, using Python/*Scikit‐learn*. Then *EBayes* = 15,000 *Gibbs* samples with $\theta_{\text{Gibbs}}\left(\hat{\mu },\hat{k}\right)$ were generated, which $\hat{\mu }$ implies to regularization and $\hat{k}$ to penalty parameters. The prior distribution was chosen to be uninformative to mildly informative.

## Author Contributions

M.B.S. was responsible for the problem statement, performing the experiments, data collection and analysis, and writing original draft; A.B. was responsible for the supervision of the experiments and draft editing; S.D. was responsible for the resources and provided the improved marker data with the physical map; N.S. and A.P.K. supported data collection; M.K. supported data collection and editing manuscript draft; J.L. was responsible for data analysis and interpretation, study supervision, and funding acquisition.

All authors have read and agreed to the published version of the manuscript.

## Conflicts of Interest

The authors declare no conflicts of interest.

### Peer Review

The peer review history for this article is available at https://www.webofscience.com/api/gateway/wos/peer‐review/10.1002/pld3.70036.

## Supporting information


**Data S1** Peer review.

**Data S2** Supporting information.

## Data Availability

The data that support the findings of this study are available from the corresponding author upon reasonable request.
