# Supplementary material for: The Significant Effects of Threshold Selection for Advancing Nitrogen Use Efficiency in Whole Genome of Bread Wheat
Source: Plant Direct. 2025 Jan 21;9(1):e70036. doi: 10.1002/pld3.70036 (PMC11750810; doi:10.1002/pld3.70036)
Supplement: Supplementary file 2 — Data S2 Supporting information. [file PLD3-9-e70036-s002.pdf]

Manuscript: Plant Direct-2024-01430 (The significant effects of threshold selection for advancing nitrogen use efficiency in whole genome of bread wheat)

Supplementary Materials:

Software description:

R/*agricolae:*

Original idea was presented in the thesis "A statistical analysis tool for agricultural research" to obtain the degree of Master on science, National Engineering University (UNI), Lima-Peru. Some experimental data for the examples come from the CIP and others research. *Agricolae* offers extensive functionality on experimental design especially for agricultural and plant breeding experiments, which can also be useful for other purposes. It supports planning of lattice, Alpha, Cyclic, Complete Block, Latin Square, Graeco-Latin Squares, augmented block, factorial, split and strip plot designs. There are also various analysis facilities for experimental data, e.g. treatment comparison procedures and several non-parametric tests comparison, biodiversity indexes and consensus cluster (the canonical link https://CRAN.R-project.org/package=agricolae to use).

R/*bootstrap:*

Software (bootstrap, cross-validation, jackknife) and data for the book "An Introduction to the Bootstrap" by B. Efron and R. Tibshirani, 1993, Chapman and Hall. This package is primarily provided for projects already based on it, and for support of the book. New projects should preferentially use the recommended package *bootstrap* (the URL link https://gitlab.com/scottkosty/bootstrap to use).

R/*rrBLUP:*

Software for genomic prediction with the RR-BLUP mixed model (Endelman 2011, <doi:10.3835/plantgenome2011.08.0024>). One application is to estimate marker effects by ridge regression; alternatively, BLUPs can be calculated based on an additive relationship matrix or a Gaussian kernel.

R/*mlmm.gwas:*

Pipeline for Genome-Wide Association Study using Multi-Locus Mixed Model from Segura V, Vilhjálmsson BJ et al. (2012) <doi:10.1038/ng.2314>. The pipeline include detection of associated SNPs with MLMM, model selection by lowest eBIC and raw *p-value* threshold, estimation of the effects of the SNPs in the selected model and graphical functions.

R/*Synbreed*:

This package provides a framework for the analysis of genomic prediction data (Genomic Selection, GWAS, QTL-mapping) within an open source software (the URL link https://synbreed.r-forge.r-project.org/ to use).

R/*fdrtool*:

Estimates both tail area-based false discovery rates (FDR) as well as local false discovery rates (fdr) for a variety of null models (p-values, z-scores, correlation coefficients, t-scores). The proportion of null values and the parameters of the null distribution are adaptively estimated from the data. In addition, the package contains functions for non-parametric density estimation (Grenander estimator), for monotone regression (isotonic regression and antitonic regression with weights), for computing the greatest convex minorant (GCM) and the least concave majorant (LCM), for the half-normal and correlation distributions, and for computing empirical higher criticism (HC) scores and the corresponding decision threshold (the canonical link https://CRAN.R-project.org/package=fdrtool to use).

R/*fdrestimation*:

The user can directly compute and display false discovery rates from inputted *p-values* or z-*scores* under a variety of assumptions. p.fdr() computes FDRs, adjusted *p-values* and decision reject vectors from inputted *p-values* or z-*values*. get.pi0() estimates the proportion of data that are truly null. plot.p.fdr() plots the FDRs, adjusted p-values, and the raw *p-values* points against their rejection threshold lines (the canonical link https://CRAN.R-project.org/package=FDRestimation to use).

R/Bioconductor/*qvalue*:

This package takes a list of p-values resulting from the simultaneous testing of many hypotheses and estimates their *q-values* and local FDR values. The *q-value* of a test measures the proportion of false positives incurred (called the false discovery rate) when that particular test is called significant. The local FDR measures the posterior probability the null hypothesis is true given the test's *p-value*. Various plots are automatically generated, allowing one to make sensible significance cut-offs. Several mathematical results have recently been shown on the conservative accuracy of the estimated *q-values* from this software. The software can be applied to problems in genomics, brain imaging, astrophysics, and data mining (the URL link http://github.com/jdstorey/qvalue to use).

R/Bioconductor/*twilight*:

In a typical microarray setting with gene expression data observed under two conditions, the local false discovery rate describes the probability that a gene is not differentially expressed between the two conditions given its corresponding observed score or *p-value* level. The resulting curve of *p-values* versus local false discovery rate offers an insight into the twilight zone between clear differential and clear non-differential gene expression. Package 'twilight' contains two main functions: Function twilight.pval performs a two-condition test on differences in means for a given input matrix or expression set and computes permutation based *p-values*. Function twilight performs a stochastic downhill search to estimate local false discovery rates and effect size distributions. The package further provides means to filter for permutations that describe the null distribution correctly. Using filtered permutations, the influence of hidden confounders could be diminished (the URL link http://compdiag.molgen.mpg.de/software/twilight.shtml to use).

Python/*Scikit-learn*:

Model selection and evaluation (the URL link https://scikit-learn.org/stable/model_selection.html to use).
