# Supplementary material for: The Significant Effects of Threshold Selection for Advancing Nitrogen Use Efficiency in Whole Genome of Bread Wheat
Source: Plant Direct. 2025 Jan 21;9(1):e70036. doi: 10.1002/pld3.70036 (PMC11750810; doi:10.1002/pld3.70036)
Supplement: Supplementary file 1 — Data S1 Peer review. [file PLD3-9-e70036-s001.pdf]

Plant Direct
Open Access

**The significant effects of threshold selection for advancing nitrogen use efficiency in whole genome of bread wheat**

Mohammad Bahman Sadeqi [1], Agim Ballvora [1], Said Dadshani [2], Md. Nurealam Siddiqui [1,3], Mohammad Kamruzzuman [1], Ahossi Patrice Koua [1], Jens Léon [1,4]

**Decision Letter Round 1:**

July 22, 2024

Agim Ballvora
University of Bonn
Katzenburgweg 5
Bonn 53115
Germany

RE: Significant threshold selection for nitrogen use efficiency in whole genome of bread wheat

Dear Dr. Ballvora:

Thank you for submitting to Plant Direct. All required reviews have been returned and we have now finished our evaluation of your manuscript. In light of the reviewers' and editor's comments, further revisions are needed before the paper can be accepted for publication in Plant Direct.

Please view the editors' and reviewers' comments below and use their suggestions as a guide while you work on your revision.

When uploading the revised version of this article, please be sure to include the following:

-A word document that contains your response to the reviewers. You should respond to each reviewer comment and note the changes made to the manuscript. If you do not agree with a reviewer's comment and choose not to make a suggested revision, please explain why. Please try to provide as complete an answer as possible to each reviewer's criticisms.
-A tracked changes document with each change highlighted
- A clean version of the latest version of the manuscript

I would like to apologize to the authors for this lengthy review time. It has been very hard to find reviewers.

To upload your revision, please click the link below.
https://plantdirect.msubmit.net/cgi-bin/main.plex?el=A2Lr1gJ6A6nLr2I4A9ftdgPn9P1qhfain4ECaOOwCKQZ

In order to provide as timely a service as possible, we ask that your revision is resubmitted within three months after receipt of this request. If an extension is needed, please send a request, along with a brief explanation, to the editorial office at plantdirect@wiley.com .

Please note that, in addition to publishing reviewer comments, the author's responses to review comments will also be published alongside the final version of the paper. If you would not like the author's responses to be published, please contact the editorial office at plantdirect@wiley.com .

Thank you very much for giving us an opportunity to review your work. I look forward to receiving the next version.

Sincerely,

Ruben Rellan-Alvarez

Esther van der Knaap

Editor, Plant Direct

------------------------------------------------------------------------------- Editor comments:
I would like to apologize to the authors for this lengthy review time. It has been very hard to find reviewers.

------------------------------------------------------------------------------- Reviewer comments:
Reviewer #1:

Genome-wide association studies are an important tool used by breeders and geneticists to understand the genetic architecture of complex traits in a considered population. In breeding programs, GWAS for major genes can inform the use of markers for trait prediction. A lack of major QTL contributing to trait variation may suggest a focus on genome-wide prediction, while detection of a few major QTL associated

with most of trait variation may suggest a focus on marker development and marker-assisted selection. To interpret these results, it's important to understand if the GWAS performed is appropriately powered -- is the lack of "significant" QTL a function of a polygenic trait architecture, or is the study just underpowered? Alternative methods for informing LOD thresholds for GWA studies may improve our ability to interpret the results of these studies, and clarify the implications of GWA studies for the use of genetic data in breeding for the considered traits.

In "Significant threshold selection for nitrogen use efficiency in whole genome of bread wheat", the authors consider a data panel of wheat varieties assayed for nitrogen use efficiency and genotyped with genome-wide SNP markers, then approach GWA analyses in this population with differing modeling and threshold determination approaches. In winter wheat, NUE is an important trait that is receiving increased interest due to the potential environmental benefit of wheat in rotation with corn and soybean. The study is therefore relevant to the broader community. Unfortunately, the quality of writing and consistent mistakes in grammar make an understanding of the details of the analyses and results difficult. For this reason, I do not believe I am able to appropriately evaluate the manuscript at this moment. Due the relevance of the research, I would encourage the authors to revise the manuscript to improve the overall quality of the writing and grammar prior to re-submission.

As part of their re-submission, the authors should further elaborate on the methods used to collect the nitrogen use efficiency data. This should include details such as the germplasm used (where were the genotypes sourced from? are they landraces or elite varieties? hard/soft and spring/winter wheat?), and details on the experimental design (nitrogen rates, plot size, approximate planting dates) and phenotype collection (how was NUE specifically assayed? what is straw nitrogen yield specifically and how was it collected?).

Reviewer #2:

Dear Authors,

Your prepared manuscript related to biostatistical studies of bread wheat genome is a valuable work. However, the following information should be added to remove its slight deficiencies:
1.Change its title as "The significant effects of threshold selection for advancing nitrogen-use efficiency in whole genome of bread wheat".
2.Prepare a glossary or wordlist of all abbreviations (that used more than one time in the text) after the Abstract and before the Introduction.
3.Use complete wordings of each abbreviation when it is used at the first time in the text content of manuscript.
4.Use numerical numbers for the equations you explained in the text content.
5. Review the whole text content to remove any typing mistake.

Plant Direct
Open Access

**Decision Letter Round 2:**

December 25, 2024

Dr. Agim Ballvora
Rheinische Friedrich-Wilhelms-Universitat Bonn
Katzenburgweg 5
Bonn 53115
Germany

MSID: 2024-01430R1
MS TITLE: The significant effects of threshold selection for advancing nitrogen use efficiency in whole genome of bread wheat

Dear Dr. Agim Ballvora:

I am pleased to inform you that your manuscript "The significant effects of threshold selection for advancing nitrogen use efficiency in whole genome of bread wheat" has been accepted for publication in Plant Direct.

Your article will appear online in the next available issue of Plant Direct. To ensure your article gets published as quickly as possible, please pay attention to the steps detailed below. We have found that most of the delays happen at this stage, especially at the payment stage, so please respond as quickly as possible when prompted.

License Agreement: Once your article has been accepted it will move to Production and undergo admin and file checking - you may receive an email with any queries we have at this stage. When all required items are received by the publisher and queries resolved, the corresponding author will receive an email from Wiley's Author Services system which will ask them to log on at https://authorservices.wiley.com/bauthor and will present them with the appropriate license for completion. Your article cannot be published until both the signed license agreement and payment of the article fee have been received.

Payment of the Open Access Article Publication Fee: All articles published in Plant Direct are fully open access: immediately and freely available to read, download and share. Plant Direct charges a publication fee to cover the publication costs. The corresponding author for this manuscript should have already received a quote from the payments team (cs-openaccess@wiley.com) with the estimated article publication fee; please email cs-openaccess@wiley.com if this has not been received. The corresponding

author should log on to the Wiley Author Services site, where the publication fee can be paid by credit card or an invoice. Pro Forma can also be requested. Payment of the publication charge must be received before the article will be published online.

Proofs: You will have the opportunity to look over your paper once more when you receive the author proofs for your article. The proofs will be with you in approximately two weeks. Please note that, in addition to publishing reviewer comments, the author's responses to review comments will also be published alongside the final version of the paper. If you would not like the author's responses to be published, please contact the editorial office at plantdirect@wiley.com .

Promotion of your article: You can help your research get the attention it deserves! Check out Wiley's free Promotion Guide for best-practice recommendations for promoting your work at www.wileyauthors.com/eeo/guide. And learn more about Wiley Editing Services which offers professional video, design, and writing services to create shareable video abstracts, infographics, conference posters, lay summaries, and research news stories for your research at www.wileyauthors.com/eeo/promotion.

Thank you again for your contribution to Plant Direct. If you have any questions, feel free to contact the editorial office at plantdirect@wiley.com .

Sincerely,

Ruben Rellan-Alvarez

Esther van der Knaap

Editor, Plant Direct

-------------------------------------------------------------------------- Editor comments
Dar authors,

please address the small comments from the reviewer before final acceptance. Sorry for the lengthy review process.

-------------------------------------------------------------------------- Reviewer comments:
Reviewer #2:

Authors

Please add my suggestive and brief conclusion at the end of manuscript (before Acknowledgment):

Conclusion
Utilizing modern statistical optimization (to assess stability and performance) for finding significant FDR threshold is a necessity. It is feasible to improve the reliability of FDR thresholding by incorporating neural network algorithms and increase the chances of identifying true genetic relations of bread wheat while minimizing the risk of false positives.

Also insert the wordings of the following terms in parenthesis:
R/rrBLUP
GRM
IBS
R/mlmm.gwas
R/fdrtool

Good luck
One of the Reviewers

**Response to Reviewer:**

**Manuscript: Plant Direct-2024-01430 (The significant effects of threshold selection for advancing nitrogen use efficiency in whole genome of bread wheat)**

**Response to reviewers' comments**

We are very grateful for the reviews provided by the editors and each of the reviewers of this manuscript. It was your valuable and insightful comments that led to possible improvements in the current version. We have carefully considered the comments and tried our best to address every one of them. We have subjected our manuscript to extensive editing of English language and it has been completely rewritten again. We hope the manuscript after careful revisions meet your high standards. The authors welcome further constructive comments if any. Please, below we provided the point-by-point responses to the comments in blue.

**First Reviewer:**

**Comment from reviewer:** As part of their re-submission, the authors should further elaborate on the methods used to collect the nitrogen use efficiency data. This should include details such as the germplasm used (where were the genotypes sourced from? are they landraces or elite varieties? hard/soft and spring/winter wheat?), and details on the experimental design (nitrogen rates, plot size,

approximate planting dates) and phenotype collection (how was NUE specifically assayed? what is straw nitrogen yield specifically and how was it collected?).

**Authors' Response:** Thank you for this excellent observation. We included the requested details on field experiment in Method: phenotypic dataset. The order and sequence of the sentences must be coherent and connect to give the reader clear orientation. Thus, we have subjected our manuscript to extensive editing of English language and it has been completely rewritten again.

**Second Reviewer:**

**Comment from reviewer:** Change its title as "The significant effects of threshold selection for advancing nitrogen-use efficiency in whole genome of bread wheat".

**Authors' Response:** Thank you very much for this suggestion. In this manuscript, due to focus on different thresholding methods, it would be interesting to change the title to your suggestion.

**Comment from reviewer:** Prepare a glossary or wordlist of all abbreviations (that used more than one time in the text) after the Abstract and before the Introduction.

**Authors' Response:** Thank you for pointing this out. A list of all abbreviations has been provided before introduction part.

**Comment from reviewer:** Use numerical numbers for the equations you explained in the text content.

**Authors' Response:** Thank you for pointing this out. In this version every equation has specific number.

**Comment from reviewer:** Review the whole text content to remove any typing mistake.

**Authors' Response:** Thank you for this excellent observation. We have subjected our manuscript to extensive editing of English language and it has been completely rewritten again.
